# Metabolomics in oncology

**DOI:** 10.1002/cnr2.1795

**Published:** 2023-02-21

**Authors:** Gurparsad Singh Suri, Gurleen Kaur, Giuseppina M. Carbone, Dheeraj Shinde

**Affiliations:** ^1^ Department of Biological Sciences California Baptist University Riverside California USA; ^2^ Institute of Oncology Research (IOR) Universita’ della Svizzera Italiana (USI) Bellinzona Switzerland

**Keywords:** biomarker, cancer, metabolic reprogramming, metabolism, metabolomics

## Abstract

**Background:**

Oncogenic transformation alters intracellular metabolism and contributes to the growth of malignant cells. Metabolomics, or the study of small molecules, can reveal insight about cancer progression that other biomarker studies cannot. Number of metabolites involved in this process have been in spotlight for cancer detection, monitoring, and therapy.

**Recent Findings:**

In this review, the “Metabolomics” is defined in terms of current technology having both clinical and translational applications. Researchers have shown metabolomics can be used to discern metabolic indicators non‐invasively using different analytical methods like positron emission tomography, magnetic resonance spectroscopic imaging etc. Metabolomic profiling is a powerful and technically feasible way to track changes in tumor metabolism and gauge treatment response across time. Recent studies have shown metabolomics can also predict individual metabolic changes in response to cancer treatment, measure medication efficacy, and monitor drug resistance. Its significance in cancer development and treatment is summarized in this review.

**Conclusion:**

Although in infancy, metabolomics can be used to identify treatment options and/or predict responsiveness to cancer treatments. Technical challenges like database management, cost and methodical knowhow still persist. Overcoming these challenges in near further can help in designing new treatment régimes with increased sensitivity and specificity.

## 1 INTRODUCTION

Metabolomics includes the systematic identification and quantification of metabolic products from the human body. In this review, we emphasize its relevance and potential applications in the oncology field.

Cancer is one of the leading causes of mortality worldwide and a key deterrent to increasing global life expectancy.^1^ According to WHO estimates for 2019, cancer is the main cause of death for adults aged below 70 in most countries.^2^ Cancer cells have a faulty metabolism causing uncontrolled proliferation. This altered metabolism generates unique metabolic characteristics that can be used to aid in early cancer detection, personalized treatment, and/or gauge therapeutic response.^3^, ^4^

Metabolic changes in cancer patient due to treatment, nutrition, and exercise can influence cancer outcomes and patient quality of life.^5^, ^6^ Metabolites can be evaluated in several body fluids such as blood, plasma and urine and therefore represent a potential non‐invasive tool for the management of cancer patients, eventually providing a novel set of diagnostic biomarkers for tumor status and progression. Moreover, its study can support the management of the anticancer treatment response at an individualized level and also predict failures.^7^

Biochemical processes and metabolic pathways can be described in details using metabolomics rather than standard clinical laboratory procedures.^8^ In metabolomics, metabolites such as monosaccharides, amino acids, small lipids, co‐factors, vitamin B complexes, energy cycle intermediates, nucleotides, exogenous xenobiotics and more are measured and studied comprehensively.^9^, ^10^ Biomass, energy and redox balance all play a key role in cell metabolism, which is essential for life. There have been numerous studies demonstrating the importance of metabolic reprogramming in a variety of disorders, including cancer, diabetes, cardiovascular disease, and neurological disease.^11^, ^12^ In order to uncover the underlying causes of disease and devise new treatments, understanding metabolism is a necessity.

The objective of this article is to give a general overview on the current and future prospects on metabolomics and its role in cancer detection, monitoring and treatment. Here, we discuss recent developments in metabolomics and then emphasize on the clinical applications of metabolomics.

## 2 REPROGRAMMING OF CANCER CELL METABOLISM

Metabolic reprogramming helps cancer cells to survive and proliferate during the course of cancer development. The enhanced growth and proliferation in malignant cells require an increased amount of energy in the form of ATP and other co‐factors. This increased demand for resources is fulfilled through alteration of flux via multiple metabolic pathways. Altered Glycolysis and glucose metabolism (Warburg effect) is the most well‐known and studied pathways in cancer metabolism. Over time multiple other pathways have been found to be altered in cancer cells for example lipid metabolism pathway, glutamine metabolism pathway, amino acid metabolism, citric acid cycle, fatty acid oxidation, one‐carbon metabolism etc.^13^ The reprogramming in theses pathways is complex and involves multiple factors. Also depending on the cancer type the reprogramming occurs in various degrees and in contexts with the microenvironmental conditions, providing required plasticity to cancer cell.

In this review we have tried to differentiate the metabolic state of cancer cells depending on the stages of cancer progression. For cancer cells to proliferate, invade, and metastasize they need to acquire a different metabolic state. It can broadly be classified into 3 stages. (A) Tumor micro‐environments are typically acidic and hypoxic with a distinct nutrient composition compared to normal tissues, forcing cancer cells to adapt to such conditions in order to survive. (B) During invasion, for survival in blood vessels, cancer cells must reprogram their metabolic state, allowing for anchorage‐independent growth. (C) Lastly, when cancer cells colonize other organs, they must adapt to a completely new metabolic environments compared to primary sites in order to grow.^14^, ^15^ Understanding the mechanisms underlying this metabolic reprogramming can help in identification of new therapeutic targets for cancer.

### 2.1 Metabolic reprogramming and tumor microenvironment

**FIGURE 1:**
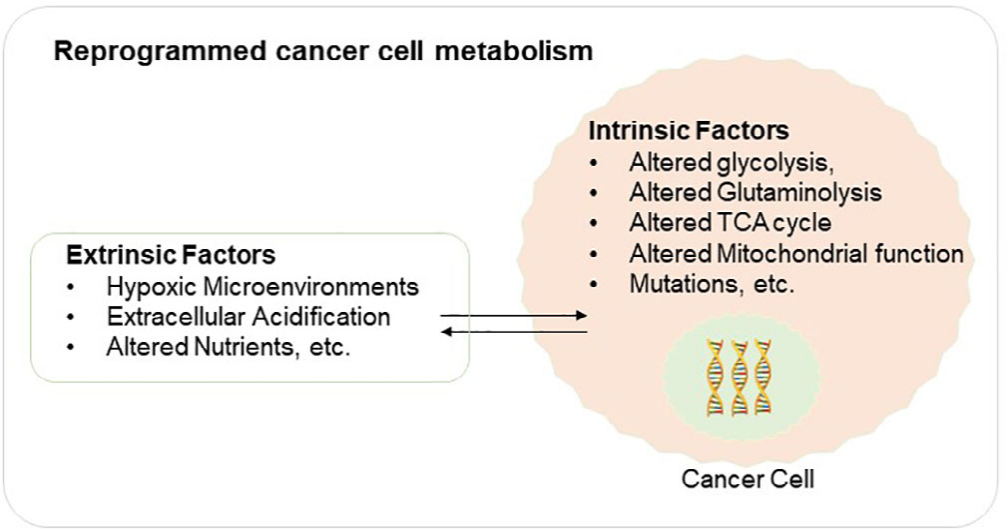
Schematics showing reprogrammed cancer cell metabolism. Extrinsic factors include hypoxic microenvironments, acidic conditions, altered nutrients while Intrinsic factors include gene mutations, oncogene deregulations. Tricarboxylic acid (TCA)

Tumor microenvironments have altered metabolic mechanism compared to normal tissues. This metabolism is influenced by number of intrinsic and extrinsic factors (Figure 1). The classic example of cell‐intrinsic factor is altered glycolysis (also known as Warburg effect),^16^ a fast glycolysis event due to the necessity of malignant proliferation. Lack of oxygen in the local tumor microenvironment is frequently caused by tumor cells high proliferative capacity and high energy requirement. However, despite the fact that glycolysis does not give as much energy as aerobic respiration, it is 100 times faster and produces the amino acids and pentose phosphates required by rapidly proliferating cancer cells.^17^, ^18^ Similarly, another most frequently altered signaling pathway in human cancer is phosphatidylinositol‐3‐kinase (PI3K)/Akt signaling pathway^19^ which along with mTOR (mammalian target of rapamycin) controls the uptake of glucose, lipids, nucleotides and amino acids.^20^, ^21^ Aberrant activation of this pathway through mutation is dominant in many tumor progressions. Other intrinsic factors include altered glutaminolysis,^22^ activated mitochondrial electron transport chain (ETC) function^23^, ^24^ altered tricarboxylic acid (TCA) cycle in many cancer types,^25^ mutations in enzymes such as isocitrate dehydrogenases 1 and 2 (IDH1, IDH2),^26^ succinate dehydrogenase (SDH) and (fumarate hydratase) FH.^27^, ^28^ On the other hand, extrinsic factors like transformed nutrient microenvironment due to heterogenous density of blood and lymphatic vessels force cancer cells for metabolic reprogramming in order to survive.^29^, ^30^ Other extrinsic factors for example hypoxia and acidification also play a critical role in metabolic reprogramming^31^, ^32^ (Figure 1). Hypoxic microenvironments lead to upregulation and stabilization of hypoxia‐inducible factors (HIFs), which are known to regulate expression of several genes (for example SNAIL, ZEB1, TWIST, matrix metalloproteinases, lactate dehydrogenase A and pyruvate dehydrogenase kinase 1^33^, ^34^, ^35^, ^36^, ^37^, ^38^) that contribute to cancer progression, including many involved in cell survival, angiogenesis, glycolysis, cancer invasion, and metastasis. Understanding these metabolic switches and their role in metabolic reprogramming can provide critical insights in designing effective treatment regimens.

### 2.2 Metabolic reprograming for anchorage‐independent growth

For cancer cells to metastasise anchorage independent growth is a requirement, wherein cancer cells must detach from extra cellular matrix, enter blood/lymphatic vessel and survive in anchorage‐independent manner. Interestingly a very small portion of circulating cancer cells are capable of doing this.^39^, ^40^ Primarily because anchorage independent growth requires metabolic reprogramming, which induce oxidative stress on the cells.^41^ A classic example of this metabolic reprogramming is reductive carboxylation of glutamine that reduces excessive reactive oxygen species (ROS) in mitochondria.^42^ This process converts glutamine derived α‐ketoglutarate (αKG) to citrate through cytosolic IDH1 enzyme. Another enzyme fatty acid synthase (FASN) is essential in maintaining the IDH1 activity^44^ thus indirectly regulating reductive carboxylation. Bueno et al., have recently showed pharmacological inhibition of FASN can prevent tumor progression.^43^ Similarly, this reductive carboxylation pathway was also found to be essential in maintaining cell proliferation under hypoxic conditions in renal cell carcinoma (RCC).^44^, ^45^ Also, pentose phosphate pathway upregulation^46^ is observed in multiple cancer types and is associated with anchorage independent growth, invasion and metastasis^47^ mainly observed in KRAS‐induced anchorage‐independent growth.^48^ Amino acid metabolism also play an important role in Anchorage‐Independent cell survival, it works by altering sphingolipid diversity through deregulation of serine, alanine, and pyruvate.^49^ In conclusion decrypting this metabolic network involved in anchorage‐independent growth can provide helpful insights in designing therapeutic strategies to prevent cancer metastasis.

## 3 METABOLIC REPROGRAMMING TO FORM METASTATIC TUMOR

One of the leading causes of death in cancer patients is metastasis. After extravasation, in order to survive cancer cells must reprogramme their metabolic status as per the new site which is distinct from that of the primary site. Thus, to adapt to this new microenvironment multiple enzymes and pathways are deregulated. For example, in metastatic breast cancer increased proline catabolism is observed via proline dehydrogenase (PRODH) upregulation compared to primary breast cancer cases,^50^ Similarly asparagine is known to increase the metastatic and invasive capabilities in breast cancer cells. It works through upregulation of asparagine synthetase (ASNS) an enzyme responsible to synthesize asparagine from aspartate.^51^ Multiple other proteins are known to be deregulated in many cancer types that support metastases for example deregulation of phosphoglycerate dehydrogenase (PHGDH),^52^ α‐ketoglutarate,^53^ monocarboxylate transporter 1 (MCT1),^54^ pentose phosphate pathway,^55^ acetyl‐CoA carboxylase (ACC)^56^ etc. Decoding these metabolic pathways and their role in metastatic can be beneficial in designing new targeted therapies against metastatic cancer types.

## 4 DRUGS TARGETING CANCER METABOLISM

Increased understanding of cancer metabolism has led to development of new drugs targeting tumor metabolism. These metabolism‐based therapies are designed to target specific metabolic pathways that are involved in tumor growth and progression. Such drugs work by either blocking the target enzyme involved in the pathway or providing a metabolic product that alters tumor metabolism. Table 1 provides a brief list of metabolism‐based anti‐cancer drugs along with their targets.

**TABLE 1:** List of metabolism‐based anti‐cancer drugs along with their targets

Drug	Target	Reference
α‐Cyano‐4‐hydroxycinnamic acid	Lactate dehydrogenase	^57^
Cinnamate	Monocarboxylate transporters	^58^
Oxamate	Lactate dehydrogenase	^59^
FX11	Lactate dehydrogenase	^60^
BPTES	Glutaminase	^61^
JHU‐083	Glutaminase	^62^
Cerulenin	Fatty acid synthase	^63^
Orlistat	Fatty acid synthase	^64^
GSK2194069	Fatty acid synthase	^65^
6‐aminonicotinamide	6‐phosphogluconate dehydrogenase	^66^
Polydatin	Glucose‐6‐phosphate dehydrogenase	^67^
A939572	Stearoyl‐CoA desaturase	^68^
Fatostatin	Sterol regulatory element‐binding protein	^69^
Soraphen A	Acetyl‐CoA carboxylase	^70^
BZ36	Stearoyl‐CoA desaturase	^71^
Fasnall	Fatty acid synthase	^72^
SB‐204990	ATP‐citrate lyase	^73^
Betulin	Sterol regulatory element‐binding protein	^74^
Triacscin C	Acetyl‐CoA synthase	^75^
Carolacton	Methylene tetrahydrofolate dehydrogenase 1 & 2	^76^
AGF347	Serine hydroxymethyltransferase 1/2	^77^
LY345899	Methylene tetrahydrofolate dehydrogenase 2	^78^
Genistein‐27	Hexokinase 2	^79^
Astragalin	Hexokinase 2	^80^
Chrysin	Hexokinase 2	^81^
Fasentin	Glucose transporters	^82^
STF‐31	Glucose transporters	^83^
Cytochalasin B	Glucose transporters	^84^
Phloretin	Glucose transporters	^85^
WZB117	Glucose transporters	^86^
Ritonavir	Glucose transporters	^87^
Koningic acid	Glyceraldehyde‐3‐phosphate dehydrogenase	^88^
Iodoacetate	Glyceraldehyde‐3‐phosphate dehydrogenase	^89^
BAY1436032	Mutant IDH1/2	^90^
Uprosertib	PI3K/Akt	^91^
Afuresertib	PI3K/Akt	^91^
Dehydroepiandrosterone	Glucose‐6‐phosphate dehydrogenase	^92^, ^93^
Ipatasertib	PI3K/Akt	^91^
AZD3965	Monocarboxylate transporters	^94^
Mitaplatin	Pyruvate dehydrogenase kinase	^95^
Dichloroacetate	Pyruvate dehydrogenase kinase	^96^, ^97^
Silybin	Glucose transporters	^98^
Resveratrol	Hexokinase 2	^99^, ^100^
3‐bromopyruvate	Hexokinase 2	^101^, ^102^
2‐deoxyglucose	Hexokinase 2	^103^, ^104^
Lonidamine	Hexokinase 2	^105^
Sorafenib	PI3K/Akt	^106^
Lonidamine	Complex II (OXPHOS)	^107^
CB‐839	Glutaminase	^108^, ^109^
TVB‐2640	Fatty acid synthase	^110^
IDH305	Mutant IDH2	^111^
FT‐2102	Mutant IDH1/2	^112^
6‐Mercaptopurine	Phosphoribosyl pyrophosphate amidotransferase	^113^
6‐Thioguanine	Phosphoribosyl pyrophosphate amidotransferase	^114^
5‐Fluorouracil	Thymidylate synthase	^115^
AG‐221 (enasidenib)	Mutant IDH1/2	^116^
AG‐881	Mutant IDH1/2	^117^
AG‐120 (ivosidenib)	IDH1	^118^
Arsenic trioxide	Complex IV (OXPHOS)	^119^
CPI‐613	Pyruvate dehydrogenase /α‐ketoglutarate dehydrogenase	^120^
Pemetrexed	Thymidylate synthase, Dihydrofolate reductase, Glycinamide ribonucleotide formyltransferase	^121^, ^122^
Capecitabine	Thymidylate synthase	^123^
Methotrexate	Thymidylate synthase, Dihydrofolate reductase	^124^

Abbreviation: IDH1, Isocitrate dehydrogenases 1; IDH2, Isocitrate dehydrogenases 2; OXPHOS, oxidative phosphorylation; PI3K, phosphatidylinositol 3‐kinase; Akt, serine/threonine‐specific protein kinases.

## 5 METABOLITE‐BASED BIOMARKERS FOR DIAGNOSIS, PROGNOSIS AND PERSONALIZED CANCER TREATMENT

It is projected by 2030 around 17 million people would die per year from cancer.^125^ Discovery of sensitive biomarkers for cancer using a tailored strategy is now a focus in cancer research and can be utilized as a detection tool for therapeutic targeting of metabolic enzymes. Early intervention in cancer treatment could lead to better outcomes if these tactics are successfully applied. In future, diagnostic and prognostic biomarkers of disease will play an important role in individualized treatment and precision medicine. Analyzing metabolic phenotypes will allow for the categorization of patients by their metabolic profiles.

For example, larkin et al.,^126^ recently showed metabolomic biomarkers in blood samples can be used to identify cancer with nonspecific symptoms.^126^ A similar study by shi et al., identified 61 differential metabolites in the plasma of children with medulloblastoma.^127^ Another study by Oshashi et al.,^128^ showed through metabolic profiling, metabolite levels differ between head and neck squamous cell carcinoma (HNSCC) and normal tissues.^128^ Such metabolite insights can significantly enhance diagnostic and prognostic outcomes in cancer patients.

Similar screening used for a large metabolomics investigation identified 15 metabolites that differed between colorectal cancer (CRC) tissue and normal tissue near the tumor.^129^ In addition to the elevation of lactate, glycerol, and glutamate linked to the Warburg effect, they found high levels of alanine, aspartate, palmitoleic acid, kynurenine, and uracil, as well as high levels of putrescine, cysteine, hypoxanthine and 2‐aminobutyrate, and low levels of myo‐inositol. In this study, four distinct CRC cohorts from Hangzhou, Shanghai, Beijing, and United States were evaluated, therefore it was believed that this panel could identify CRC in patients from different genetic origins, mutations, clinical stages, and geographic backgrounds. Moreover, CRC with a recurrence time frame of 52.9 months and better 5‐year survival rates could be distinguished from those with a shorter time frame (25.9 months) cases. From this metabolomics study, a possible predictive metabolic signature for human colorectal cancer emerged. Another study by Cacciatore et al., profiled plasma samples from 41 South African men with prostate cancer using nuclear magnetic resonance (NMR) spectroscopy.^130^ The inflammatory NMR markers, GlycA (glycoproteins containing N‐acetylglucosamine and N‐acetylgalactosamine portion), and GlycB (glycoproteins containing N‐acetylneuraminic acid portion), were quantified along with several other markers. They found the plasma of patients with aggressive and metastatic prostate cancer have extremely high levels of GlycA and GlycB supporting the use of plasma metabolome to improve the stratification of patients with prostate cancer.

In an additional study, proton magnetic resonance spectroscopy (MRS) was used to quantify 2‐hydroxyglutarate (2HG) levels in the tumors of 30 patients. Researchers observed 2HG expression correlated with IDH1 or IDH2 mutations, a common mutation in grade 2 and grade 3 gliomas.^131^ Gliomas with IDH‐1 and IDH‐2 gene mutations are more likely to have 2HG buildup.^132^ Such information could be exploited in designing more selective therapies. An example of the successful integration between clinical application and metabolomics is the study by Tenori et al.,^133^ Human epidermal growth factor receptor 2 (HER2) is used as a biomarker for precision medicine in HER2 positive metastatic breast malignancies. Its overexpression can be suppressed by the anti‐HER2 drugs. Women with metastatic breast cancer treated with paclitaxel and either anti‐HER2 medication (lapatinib) or a placebo, had their serum samples examined for metabolic profiles. Comparing the paclitaxel plus lapatinib group, researchers observed patients with HER2 positive illness were more likely to respond favorably to treatment compared to control group.^133^

Metabolomics can also be used to guide oncological surgery. Recent advances in cancer surgery have been made possible by the introduction of the biomarker‐based iKnife technology. This technology involves the intra‐operative, Rapid Evaporative Ionization Mass Spectrometry (REIMS) coupled to electrosurgical tools to allow for near real‐time characterization of margins during cautery‐led tumor dissection. Upon clinical validation of the metabolomics profiling method, surgeons may immediately be able to determine if tissue is healthy or cancerous based on the “smoke‐based” metabolomics profiling of healthy and diseased cells.^134^

### 5.1 Metabolic markers in the progression of cancer

For early detection and screening of malignant tumors, numerous new tumor markers have emerged as a result of comprehensive development in modern molecular biology techniques; nonetheless, current conventional tumor indicators lack sensitivity and specificity for the early detection of malignancies. As an alternative metabolomics can be used to analyze and validate metabolites as biomarkers for early detection of malignancies or to correctly and sensitively determine tumor progression in clinical context. An overview of metabolome investigations in different types of tumors is provided in Table 2.

**TABLE 2:** List of metabolites identified in pathological samples of cancer patients

Tumor types	Special metabolites	Metabolic pathways	References
Lung and liver cancer	Polyamine Metabolites Profiling in human plasma and urine distinguish liver from lung cancer	Polyamine metabolome pathways	^135^
Papillary thyroid micro carcinoma	Increased plasma concentrations of mannose, glucose, pyruvate, and 3‐hydroxybutyrate	Glycolysis and amino acid synthesis	^136^
Prostate Cancer	NMR spectroscopy in plasma revealed high levels of GlycA and GlycB associated with aggressive prostate cancer subtypes.	Carbohydrate portions of glycoproteins	^130^
Prostate cancer	Urine metabolomic profile of prostate cancer patients revealed increase in glutamate and pseudouridine, and decrease in glycine, dimethylglycine, fumarate and 4‐imidazole‐acetate compared with benign disease.	Branched‐chain amino acids Glucose metabolism in the TCA cycle	^137^
Bladder cancer	Urine metabolomics revealed high quantities of acetyl carnitine and adipate in cancer patients.	Metabolism of fatty acids and carnitine	^138^
Pancreatic cancer	High level of Lycocholic acid; melatonin; hippuric acid; agmatine; sphinganine; beta‐sitosterol; hypoxanthine; and spermidine; inosine and creatine	Glycine and serine metabolism; sphingolipid and bile metabolism; purine and glycine metabolism	^139^
Kidney cancer	Urine metabolomics revealed high quantities of acylcarnitines in cancer patients	Acylcarnitines	^140^
Oral squamous cell carcinoma	Glycerol and leucine production are boosted along with the production of fatty acids and steroid hormones	Fatty acid, amino acid, and glycolysis	^141^
Malignant gliomas	Metabolomics of human cerebrospinal fluid revealed high concentration of citric acid cycle components in MG	The citric acid cycle, gluconeogenesis, and pyrimidine metabolism, urea cycle	^142^
Lung cancer	Metabolomic in bronchoalveolar lavage fluid revealed lower level of Inositol, phosphoric acid, and palmitic acids in cancer patients compared to healthy individuals.	Glucose and fatty acid metabolism	^143^
Epithelial Ovarian cancer	Urinary metabolomics profiling revealed increase in (pseudouridine, N4‐acetylcytidine), (L‐histidine, imidazol‐5‐yl‐pyruvate), (3‐indolelactic acid), (3’‐sialyllactose and 3‐sialyl‐N‐acetyllactosamine) in EOC vs Benign lesions.	Metabolism of nucleotides, histidine, tryptophan, and mucin	^144^
Medullo‐ blastoma (MB)	Multiomic analysis in Cerebrospinal Fluid revealed parallel increase in genes / protein and metabolites induced by hypoxia in recurrent MB compared to benign lesions. The metabolites increased were: tryptophan, methionine, serine and lysine, (known to be induced upon hypoxia in CSF).	Amino acid and lipid metabolism	^145^

Abbreviation: NMR, Nuclear magnetic resonance; GlycA, glycoproteins containing N‐acetylglucosamine and N‐acetylgalactosamine portion; GlycB, glycoproteins containing N‐acetylneuraminic acid portion; TCA, tricarboxylic acid cycle; MG, Malignant gliomas; EOC, Epithelial Ovarian cancer; MB, Medullo‐ blastoma; CSF, Cerebrospinal Fluid.

## 6 DETECTION METHODS

There are several methods used for detection in metabolomics, including nuclear magnetic resonance (NMR), mass spectrometry (MS), metabolic flux analysis (MFA) etc. These techniques can be used alone or in combination to identify and quantify the levels of various metabolites in biological samples. Currently metabolic profiling is performed mainly by exploiting 2 principal techniques: NMR and MS. Both these techniques require small amount of sample and can identify and quantify wide range of molecules simultaneously.^146^

**FIGURE 2:**
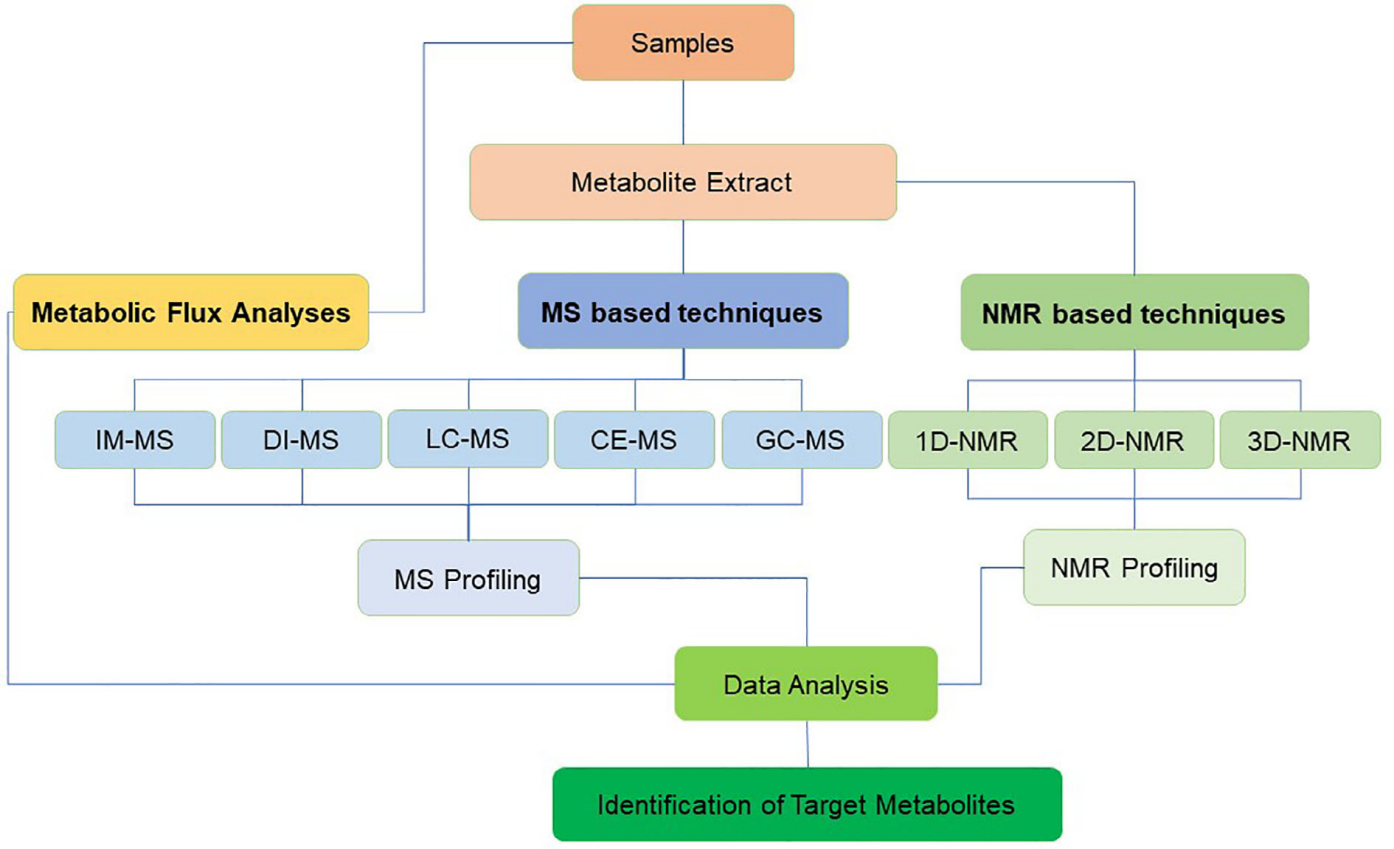
Brief classification of detection methods used in metabolomics. (NMR: nuclear magnetic resonance, 1D‐NMR: one‐dimensional nuclear magnetic resonance, 2D‐NMR: two‐dimensional nuclear magnetic resonance, 3D‐NMR: three‐dimensional nuclear magnetic resonance, MS: mass spectrometry, IM‐MS: ion mobility mass spectrometry, DI‐MS: direct injection mass spectrometry, LC–MS: liquid chromatography mass spectrometry, CE‐MS: capillary electrophoresis mass spectrometry, GC–MS: gas chromatography mass spectrometry)

NMR spectroscopy is a powerful tool for the detection of metabolites in cancer. In this technique magnetic field and radio frequency pulses are applied on the molecules to characterize the resonant frequency of that atomic nucleus. Thus, providing information about the molecular structure, motion, and chemical environment of the molecules. Hydrogen is most commonly targeted nucleus in biological samples.^147^ NMR spectroscopy can be used to identify and quantify a wide range of metabolites, including small molecules, lipids, and amino acids, in cancer cells and tissues. One of the advantages of using NMR for metabolomics in cancer is its ability to provide metabolic fingerprints that can be used to distinguish between normal and cancerous tissue.^148^ NMR spectroscopy can also be used to monitor changes in metabolite levels during cancer progression and treatment. It has also been used to study a variety of cancer types, including colon,^149^ lung,^150^ colorectal,^151^ endocrine,^152^ etc. NMR spectroscopy can also be used in conjunction with other techniques, such as mass spectrometry (MS), to provide a more comprehensive understanding of the metabolic changes associated with cancer. NMR Spectroscopy can further be broadly classified into (a) one‐dimensional, (b) two‐dimensional, and (c) three‐dimensional NMR methods (1D‐NMR, 2D‐NMR, and 3D‐NMR) (Figure 2). Limitations for NMR include initial high start‐up cost and requirement of high user skills.

On the other hand, MS is a destructive method that relies upon the generation of gas phase ions. These ions are then separated depending on their mass‐to‐charge ratio and the amount of ionization at each point in time is detected. This data can be analyzed to determine the chemical composition of the samples. One of the advantages of using MS for metabolomics in cancer is its high sensitivity and specificity, which allows for the detection of very small amounts of a wide range of metabolites.^153^ MS analysis has been used to study a variety of cancer types, and also in identification of different biomarkers in cancer.^154^ For example, Han et al., recently showed four lipid‐based biomarkers that may be used for early diagnosis in lung cancer.^155^ MS can be coupled with other analytical techniques. Based on this coupling MS can be further classified into (a) ion mobility‐MS (IM‐MS), (b) direct injection‐MS (DI‐MS), (c) liquid chromatography‐MS (LC–MS), (d) capillary electrophoresis‐MS (CE‐MS) and (e) gas chromatography‐MS (GC–MS). Such coupling provides a more inclusive understanding of the metabolic variations related with cancer.

Apart from these 2 principal techniques other techniques like metabolic flux analysis (MFA) are gaining popularity in cancer research. In this technique concurrent identification and quantification of metabolic fluxes are interpreted numerically using stoichiometric models. This permits study on huge set of reactions (e.g., transport of metabolites, anabolic reactions, catabolic reactions etc.) that define the make‐up and metabolism in malignant cells.^156^, ^157^ Figure 2 broadly classifies these detection methods. Overall, choosing and using the right techniques in detection of metabolites in cancer can provide valuable insights into the metabolic changes associated with cancer progression and treatment, and may aid in the development of new diagnostic and therapeutic strategies for cancer.

## 7 THE FUTURE OF METABOLOMICS IN ONCOLOGY

Metabolomics, encompasses a wide range of metabolite analysis, pattern identification, and statistical analysis. For prognostic or predictive interpretation of illness status, metabolic biomarkers can be widely employed in the clinical setting. Multivariate biomarkers, such as cancer fingerprint, profile or signature, should be identified using metabolomics as a biomarker discovery tool.^158^ Oncologists may soon be able to detect cancer earlier, when it is still treatable, evaluate the aggressiveness of cancer for better prognosis and treatment, and forecast therapeutic effectiveness with the use of this technology. Despite the fact that advanced analytic procedures are required, these signatures are practical and accurate. Metabolomics has been shown to be a reliable diagnostic tool for leukemia and breast cancer, respectively.^159^ Genome microarrays and serum protein profiles have been used in the past to diagnose colon and ovarian cancers, respectively. Metabolomics can be helpful in malignancies that are difficult to diagnose. Ovarian cancer has already been diagnosed using metabolomic profiles from serum.^160^

Ascitic fluid, pancreatic secretions, and bronchoalveolar or pleural fluid may all be useful in the diagnosis of cancers in future. When no other test can provide a conclusive response, metabolomics may save time, money and effort by identifying pathognomonic patterns in these fluids and validating them. Even more promising is the prospect of screening cancers through conveniently accessible bodily fluid. Development and application of metabolite spectrum databases, cross‐validation between NMR and MS metabolites, and correlation with other quantitative assays will all play a role in the future of metabolite research. Finally, the results of metabolomic analyses must be integrated with results from other omics technologies in order to characterize the whole malignant phenotypic range.

Misdiagnosis, recurrence of symptoms, and undesirable side effects can restrict the clinical efficacy of current clinical practice in illness control. Biomarkers associated with disease‐specific alterations can be examined to establish a tailored method for treating and monitoring disease development by comparing the metabolomic profiles of two or more disease phenotypes.

To date, metabolomics implementation in clinic is still not common. There are still open problems to be solved including the scalability of data interpretation and standardization of sample handling practice. The successful transition from research tool to a clinically implemented tool requires cooperation between several disciplines. To date the major problems to be solved are related to equipment design, experimental validation, standardization of methods, and data interpretability in a reproducible fashion. Strict protocols are needed to eliminate any potential biases that may arise from using metabolomics on a variety of biological fluids, including cerebrospinal fluid (CSF), urine, plasma, sweat etc. The metabolomics profile can be highly variable if the time interval between sample collection and processing is not strictly regulated. Furthermore, the quality of the data obtained is harmed by incorrect storage and repeated freeze–thaw cycles. Overcoming these hurdles can greatly benefit clinical application of metabolomics in the long run.

Metabolomics can make cancer precision medicine more feasible. Prior to moving into in vivo testing, in‐silico models can be employed to understand the effects of medicines on metabolic characteristics. Metabolomics has opened new avenues for cancer research and is already influencing cancer diagnosis and treatment in number of different ways.^161^

## 8 CONCLUSION

Although in infancy metabolomics can make a substantial impact on personalized cancer medicine. Using metabolomics, many cancer phenotypes can be accurately described with individualized metabolic markers. This can be used to identified treatment options and/or predict responsiveness to treatments. Also, it will be vital to combine the outcomes of metabolomic assessments with other omics techniques to characterized the entire spectrum of the malignant phenotypes. Technical challenges like database management, cost and methodical knowhow still persist. Overcoming these challenges in near further can help in designing new treatment régimes with increased sensitivity and specificity.

### ETHICAL STATEMENT

Ethical Approval and Consent to participate is not required for such type of studies. Also, the review has been done in accordance to ethical guidelines and that is has been performed in a responsible way, with no misconduct.

## CONFLICT OF INTEREST STATEMENT

The authors have stated explicitly that there are no conflicts of interest in connection with this article.

## AUTHOR CONTRIBUTIONS

**Gurparsad Singh Suri:** Conceptualization (equal); investigation (equal); methodology (supporting); project administration (equal); resources (equal); supervision (equal); validation (equal); visualization (equal); writing – original draft (equal); writing – review and editing (equal). **Gurleen Kaur:** Conceptualization (supporting); investigation (supporting); resources (supporting); visualization (supporting). **giuseppina carbone:** Conceptualization (equal); investigation (equal); project administration (equal); supervision (lead); writing – review and editing (equal). **Dheeraj Shinde:** Conceptualization (equal); data curation (equal); formal analysis (equal); investigation (equal); methodology (equal); project administration (equal); resources (equal); writing – original draft (lead); writing – review and editing (lead).

## Data Availability

Data sharing is not applicable to this article as no new data were created or analyzed in this study.
